# Supplementary material for: ‘That’s why I wanted him to go on dialysis’ – a qualitative inductive thematic analysis of older patients’ and their family members’ perspectives on kidney failure treatment decision-making
Source: BMC Nephrol. 2025 Jul 9;26:368. doi: 10.1186/s12882-025-04275-0 (PMC12243359; doi:10.1186/s12882-025-04275-0)

Supplementary figure 1: Distress protocol for qualitative interviews

**Pre-interview**<br>↓	• Preface all interviews with the aims of the research and the nature of topics to be covered<br>• Ensure the participant knows that participation is voluntary and they are free to withdraw at any time
**Distress**<br>↓	• If a participant indicates they are experiencing a high level of stress or emotional distress<br>OR<br>• Exhibits behaviours suggesting that the interview is stressful or emotionally distressing
**Stage 1 response**<br>↓	• Offer the participant the opportunity to pause<br>• If necessary, terminate interview and stop recording equipment<br>• Listen to interviewee and offer support in situ<br>• Be aware of boundaries and responsibilities. Avoid teaching, preaching or counselling.
**Review**<br>↓	• If participant feels able to carry on, resume interview<br>• If participant is unable to carry on, go to stage 2
**Stage 2 response**<br>↓	• Stop the interview<br>• Ask participant if there is anyone they can contact
**Follow-up**	• Researcher to attend supervision with line manager and de-brief about concerns as soon as possible<br>• Follow participant up with courtesy call (if consents)

Supplementary table 1: Consolidated criteria for reporting qualitative studies (COREQ): 32-item checklist[27]

No. Item	Guide questions/description	Reported in section
**Domain 1: Research team and reflexivity**		
Personal Characteristics		
1. Interviewer/facilitator	Which author/s conducted the interview or focus group?	Materials and methods - data collection
2. Credentials	What were the researcher's credentials? E.g. PhD, MD	Materials and methods - data collection
3. Occupation	What was their occupation at the time of the study?	Materials and methods - data collection
4. Gender	Was the researcher male or female?	Materials and methods - data collection
5. Experience and training	What experience or training did the researcher have?	Materials and methods - data collection
Relationship with participants		
6. Relationship established	Was a relationship established prior to study commencement?	Materials and methods - data collection
7. Participant knowledge of the interviewer	What did the participants know about the researcher? e.g. personal goals, reasons for doing the research	Materials and methods - data collection
8. Interviewer characteristics	What characteristics were reported about the interviewer/facilitator? e.g. Bias, assumptions, reasons and interests in the research topic	Materials and methods - data collection

Domain 2: Study design		
Theoretical framework		
9. Methodological orientation and Theory	What methodological orientation was stated to underpin the study?	Materials and methods - analysis
Participant selection		
10. Sampling	How were participants selected? e.g. purposive, convenience, consecutive, snowball	Materials and methods - sampling
11. Method of approach	How were participants approached? e.g. face-to-face, telephone, mail, email	Materials and methods - sampling
12. Sample size	How many participants were in the study?	Results (paragraph 1)
13. Non-participation	How many people refused to participate or dropped out? Reasons?	Results (paragraph 1)
Setting		
14. Setting of data collection	Where was the data collected? e.g. home, clinic, workplace	Materials and methods - data collection
15. Presence of non-participants	Was anyone else present besides the participants and researchers?	Materials and methods - data collection
16. Description of sample	What are the important characteristics of the sample? e.g. demographic data, date	Results (paragraph 2) and table 1 (participant characteristics)
Data collection		
17. Interview guide	Were questions, prompts, guides provided by the authors? Was it pilot tested?	Materials and methods - data collection (interview guide provided in supplementary appendix)

18. Repeat interviews	Were repeat interviews carried out? If yes, how many?	Materials and methods - data collection (no repeat interviews)
19. Audio/visual recording	Did the research use audio or visual recording to collect the data?	Materials and methods - data collection
20. Field notes	Were field notes made during and/or after the interview or focus group?	Materials and methods - data collection
21. Duration	What was the duration of the interviews or focus group?	Results (paragraph 3)
22. Data saturation	Was data saturation discussed?	Materials and methods - sampling
23. Transcripts returned	Were transcripts returned to participants for comment and/or correction?	No

**Domain 3: Analysis and findings**		
Data analysis		
24. Number of data coders	How many data coders coded the data?	Materials and methods - analysis
25. Description of the coding tree	Did authors provide a description of the coding tree?	Materials and methods - analysis
26. Derivation of themes	Were themes identified in advance or derived from the data?	Materials and methods - analysis
27. Software	What software, if applicable, was used to manage the data?	Materials and methods - analysis
28. Participant checking	Did participants provide feedback on the findings?	No
Reporting		
29. Quotations presented	Were participant quotations presented to illustrate the themes/findings? Was each quotation identified? e.g. participant number	Results (under thematic headings) and table 2 (illustrative quotations)

30. Data and findings consistent	Was there consistency between the data presented and the findings?	Results (under thematic headings) and table 2 (illustrative quotations)
31. Clarity of major themes	Were major themes clearly presented in the findings?	Results (under thematic headings)
32. Clarity of minor themes	Is there a description of diverse cases or discussion of minor themes?	Table 2 (illustrative quotations - subthemes)

Supplementary figure 2: Flow diagram

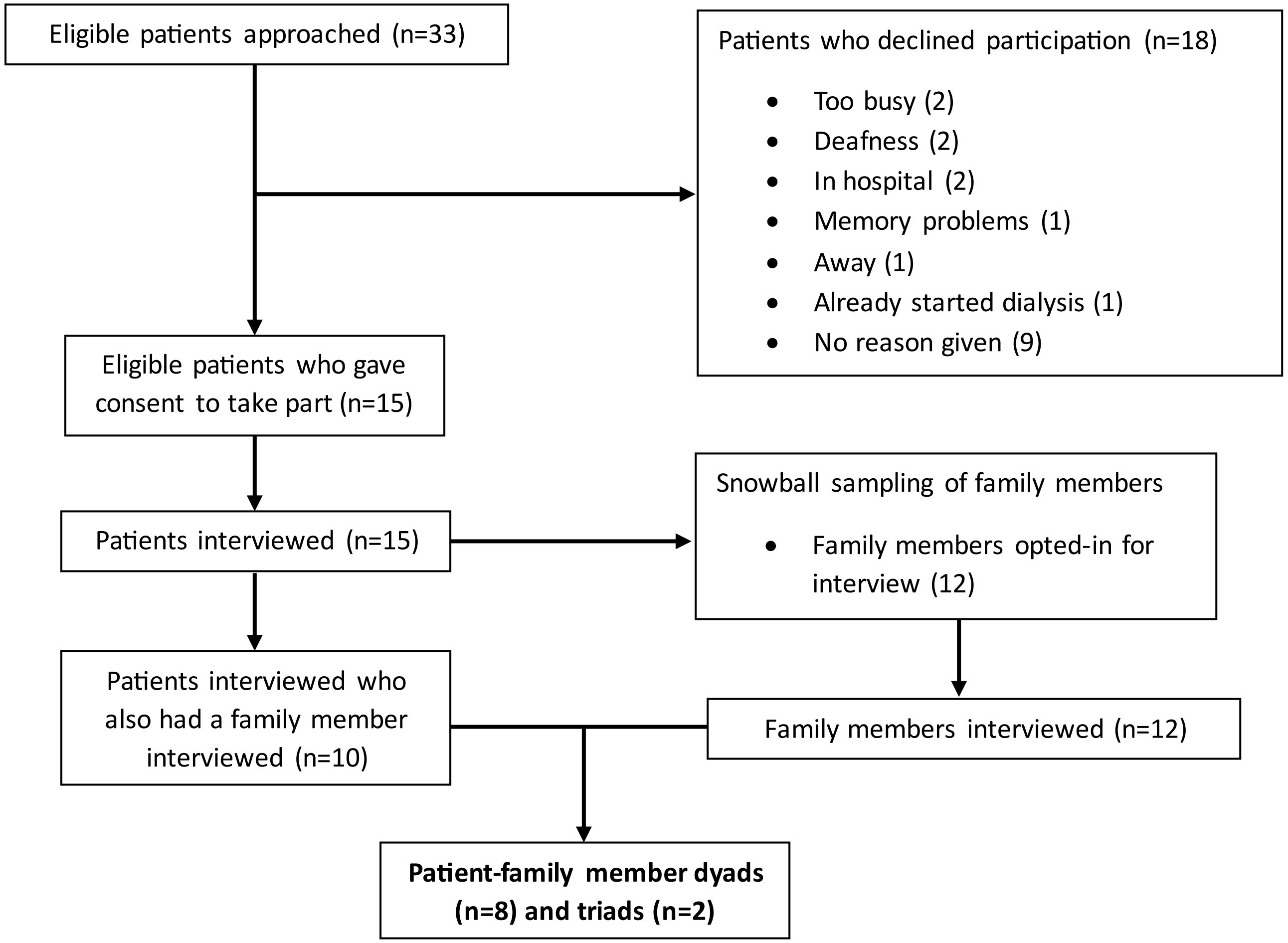

Supplement: Supplementary file 1 — Supplementary Material 1 [file 12882_2025_4275_MOESM1_ESM.pdf]
